# The Impact of COVID-19 on Plastic Surgery Residents Across the World: A Country-, Region-, and Income-level Analysis

**DOI:** 10.1007/s00266-023-03389-w

**Published:** 2023-05-30

**Authors:** Georgios Karamitros, Paraskevas Kontoes, Maria Wiedner, Sofoklis Goulas

**Affiliations:** 1 Department of Plastic Surgery, University Hospital of Ioannina, Stavrou Niarchou Avenue, 45500 Ioannina, Greece; 2 Medical School, University of Ioannina, Stavrou Niarchou Avenue, 45500 Ioannina, Greece; 3 International Society of Aesthetic Plastic Surgery, Mount Royal, NJ USA; 4 Brookings Institution, Washington DC, USA; 5 World Bank, Washington DC, USA; 6 Aletheia Research Institution, Palo Alto, CA USA; 7 Hoover Institution, Stanford University, Stanford, CA USA

**Keywords:** COVID-19, Plastic surgery training, Residency training, Global plastic surgery, Pandemic impact, Learning losses, Survey, Cross-sectional study

## Abstract

**Background:**

The COVID-19 pandemic has upended graduate medical education globally. We investigated the COVID-19 impact on learning inputs and expected learning outputs of plastic surgery residents across the world.

**Methods:**

We administered an online survey capturing training inputs before and during the pandemic and retrieved residents’ expected learning outputs compared with residents who completed their training before COVID. The questionnaire reached residents across the world through the mobilization of national and international societies of plastic surgeons.

**Results:**

The analysis included 412 plastic surgery residents from 47 countries. The results revealed a 44% decline (ranging from − 79 to 10% across countries) and an 18% decline (ranging from − 76 to across 151% countries) in surgeries and seminars, respectively, per week. Moreover, 74% (ranging from 0 to 100% across countries) and 43% (ranging from 0 to 100% across countries) of residents expected a negative COVID-19 impact on their surgical skill and scientific knowledge, respectively. We found strong correlations only between corresponding input and output: surgeries scrubbed in with surgical skill (*ρ* = *−*0*.*511 with *p < *0*.*001) and seminars attended with scientific knowledge (*ρ* = − 0*.*274 with *p* = 0*.*006).

**Conclusions:**

Our ranking of countries based on their COVID-19 impacts provides benchmarks for national strategies of learning recovery. Remedial measures that target surgical skill may be more needed than those targeting scientific knowledge. Our finding of limited substitutability of inputs in training suggests that it may be challenging to make up for lost operating room time with more seminars. Our results support the need for flexible training models and competency-based advancement.

**Level of evidence V:**

This journal requires that authors assign a level of evidence to each article. For a full description of these Evidence-Based Medicine ratings, please refer to the Table of Contents or the online Instructions to Authors http://www.springer.com/00266.

**Supplementary Information:**

The online version contains supplementary material available at 10.1007/s00266-023-03389-w.

## Introduction

The spread of the novel coronavirus forced healthcare systems globally to optimize resource allocation and re-deploy healthcare personnel to save lives [1, 2]. As a result, the increased needs of patient care eclipsed other hospital priorities and often impeded medical doctors’ training [3]. Early studies explored the impact of the COVID-19 pandemic on residents’ training [4–7]. Residents’ education in surgical specialties in particular is found to be more severely impacted by the pandemic [8–17]. Recent studies have revealed substantial learning losses among plastic surgery residents in specific regions [18–22]. In this study, we investigate the impact of the COVID-19 pandemic on learning outcomes of plastic surgery residents at the country and continent level worldwide.

Our cross-sectional survey study goes beyond previous research in four important ways. First, we target plastic surgery residents across the world, while previous studies have explored the impact of COVID-19 at a national or a regional level. Our analysis makes comparisons between countries and provides benchmarks for national strategies of learning recovery. Second, we investigate the impact of the pandemic directly on expected learning outcomes, overcoming the challenge of identifying the intermediate relation between learning inputs, such as surgeries and seminars, and outputs, such as surgical competence. Third, the data collection for our study began two years after the pandemic had started, giving us the opportunity to capture a rather complete picture of the impact of COVID-19 on plastic surgery residents across the world. According the CDC, prior pandemics lasted 1–2 years on average [23]. Fourth, our analysis provides two contextual benchmarks of pandemic-related impact: each country’s COVID-19 disease burden and its income level. These benchmarks allow us to investigate how the pandemic severity and resource availability may contribute to the magnitude of resident learning losses during COVID-19.

## Methodology

We combined data from multiple sources. First, we collected survey responses from plastic surgery residents across the world regarding their learning inputs and outputs prior to and during the COVID-19 pandemic. Second, we obtained data on each country’s COVID-19 cases and COVID-19-related deaths per million through 2021 from the Institute for Health Metrics and Evaluation (IHME) [24]. The severity of the COVID-19 pandemic in each country, measured by COVID-19 cases and deaths, may be associated with the operational pressure on local healthcare systems and any interruptions in resident training. Third, we retrieved each country’s classification in economic development from the World Bank [25]. The financial context in each country is likely to influence the operational resilience of healthcare systems and consequently the level of disruption in resident training during COVID-19.

### Survey Data

We developed a survey to capture demographics, reported changes in surgeries and seminars attended prior to and during the COVID-19 pandemic, and expected impact on surgical skill and scientific knowledge at the end of the training program due to the pandemic of plastic surgery residents across the world. The survey was administered automatically through an online link in English between January 10th and February 6th, 2022.

We followed Aucejo et al. in directly asking individuals for their expected learning outcomes with and without COVID-19 [26]. The responses allowed us to directly calculate the resident-level subjective treatment effect. Our approach builds on an established literature that uses subjective expectations on education outcomes to understand decision making under uncertainty [27–29]. The validity of our methodology relies on the assumption that residents have well-formed expectations regarding their learning outcomes in both in a reality with the COVID-19 pandemic and in a version of reality without the pandemic. This study was approved by the Institutional Review Board at Stanford University and followed the STROBE reporting guidelines [30].

We identify key learning inputs for plastic surgery residents: surgeries participated/scrubbed in and seminars attended. Residents were asked to report the number of surgeries and seminars per week or month before and during the COVID-19 pandemic. For each learning input, we calculated the percentage change in the number of surgeries and seminars attended per week, respectively, between prior to and during the pandemic. This information allowed us to understand the severity of the pandemic-related disruption in training inputs across the world. We focused on two main learning outcomes: surgical skill and scientific knowledge. We explicitly asked residents whether the impact of the pandemic on their surgical skill and scientific knowledge has been *significantly negative*, *slightly negative*, *zero*, *slightly positive*, or *significantly positive* relative to residents who completed their training prior to the COVID-19 pandemic. For each leaning outcome, we created binary variable that takes the value one when the respondent replied slightly or significantly less/negative impact.

With the help of the International Society for Aesthetic Plastic Surgery (ISAPS) we reached plastic surgery residents around the world. ISAPS is the leading professional body for board-certified plastic surgeons with a network of residents in more than 100 countries. The survey link was disseminated by 63 associations of plastic surgeons, including ISAPS, to their resident members via email and social media. The survey questions and the dissemination strategy are reported in Supplementary Appendix. All residents in plastic surgery programs in training when the pandemic started in early 2020 were eligible to participate in the survey. From administrative sources, we inferred that ISAPS had 1314 plastic surgery members in 2022. If half of them were in training during the pandemic, the maximum potential sample we could have would be around 657 residents.

## Results

### Demographics

A total of 664 plastic surgery residents responded to the survey request. Two hundred and fifteen residents did not complete the survey.^1^ Eleven responses were excluded from respondents who were not in training during the pandemic. Six duplicate responses were dropped. Twenty countries with single responses were excluded. The analytic sample included 412 respondents from 47 countries. Table 1 presents summary statistics of characteristics of participants and their training settings.

**Table 1:** Resident characteristics

	Mean
Female sex (%)	41.7
*Race (%)*	
White	61.4
Asian	25.0
Multi-racial	5.6
Black	2.4
Other	5.6
Mean age (yr)	32.1
Have dependents (%)	31.0
International medical school graduate (%)	16.3
Prior general surgery training up to 2 years (%)	63.0
Prior plastic surgery training (%)	42.1
Mean training duration (yr)	4.7
*Year of training (%)*	
PGY-1	17.0
PGY-2	21.6
PGY-3	21.4
PGY-4	20.4
PGY-5+ *Hospital type (%)*	19.7
University	59.5
Community	18.7
Tertiary	13.1
Private	5.8
Military	2.9
Residents in hospitals treating COVID-19 patients (1=yes)	91.3
Residents redeployed to COVID-19 wards (1=yes)	45.9
*N*	412

This table reports mean values of respondent characteristics

*PGY:* postgraduate year

Females represent 42% of respondents. The majority (61%) of participants are non-Hispanic white. Sixty-three percent have prior general surgery experience of up to 2 years, while 42% of residents have had plastic surgery training prior to their residency. Our sampled residents are roughly equally distributed in PGY 1 through 5+. Nearly 60% of participants work in university-affiliated hospitals, followed by community (18.7%) and tertiary (13.1%) healthcare centers. More than 90% of respondents worked in a hospital that treated COVID-19 patients, while roughly 46% of those were redeployed to COVID-19 wards.

### Learning Inputs

**Fig. 1:**
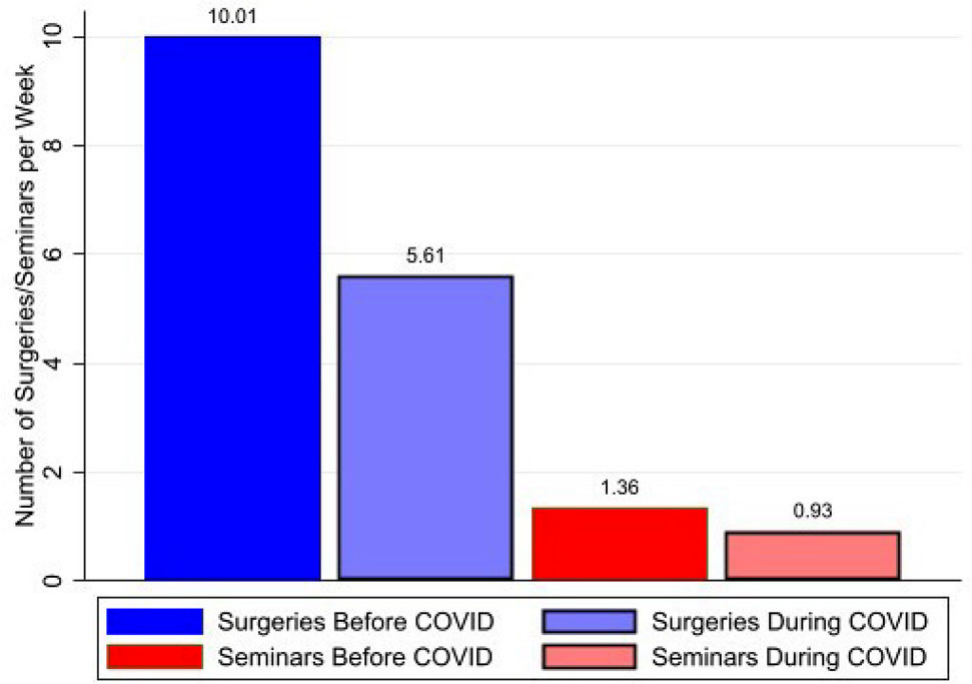
Reported Surgeries and Seminars before and during COVID-19. *Notes:* This figure shows the average number of surgeries (left two columns) and seminars (two right columns) before and during to COVID-19

Figure 1 shows the average number of surgeries and seminars plastic surgery residents attended per week prior to and during the COVID-19 pandemic. The average number of surgeries declined from 10.01 to 5.61 per week, a 44% decrease. At the same time, the number of seminars decreased from 1.36 to 0.93 per week, an 18% decline.

**Fig. 2:**
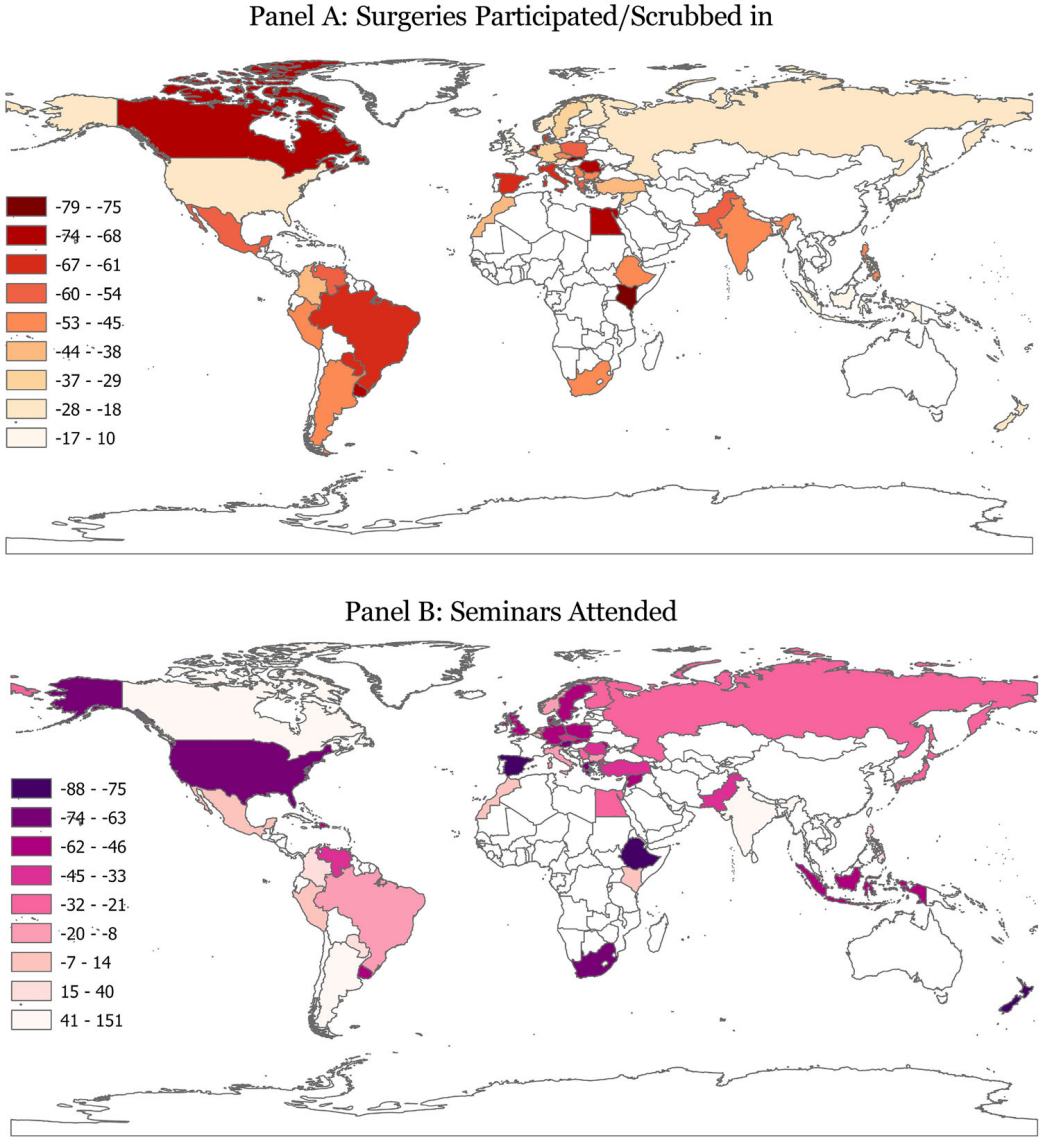
Reported Changes in Learning Inputs during COVID-19 by Country Panel A: Surgeries Participated/Scrubbed in. Panel B: Seminars Attended. *Notes:* This map shows the percentage change between prior to and during the COVID-19 pandemic of surgeries residents participated/scrubbed in (Panel A) and seminars attended (Panel B) by country. Darker shades reflect more negatively impacted countries.

Table 2 shows the percentage change in surgeries and seminars attended between prior to and during the pandemic by respondents in each country (also shown in Figure 2). Respondents from every country except for the Dominican Republic report a decrease in the number of surgeries they scrubbed in. Trainees from the Dominican Republic report that they participated in 10% more surgeries during the COVID-19 pandemic. Residents from Moldova, Japan, and the UK report the least decrease in their operation room time: − 10%, − 12%, and − 12%, respectively. On the other extreme, residents from Canada, Uruguay, Kenya, and Slovakia report that their surgical training decreased more than 70%.

**Table 2:** Changes in surgeries and seminars attended during COVID-19 by country

Country	Reported change in				N	COVID-19 cases (per million)	COVID-19 deaths (per million)	Income
	Surgeries		Seminars					
	Rank	%	Rank	%				
Dominican Rep.	1	10	25	− 26	10	37,295	378	UMI
Moldova	2	− 10	40	− 53	5	114,927	3139	LMI
Japan	3	− 12	22	− 22	9	13,984	148	HI
UK	4	− 12	10	0	5	191,647	2627	HI
Indonesia	5	− 13	34	− 50	2	15,473	523	LMI
South Korea	6	− 16	29	− 35	12	12,260	109	HI
Finland	7	− 18	19	− 21	4	48,880	309	HI
New Zealand	8	− 19	46	− 76	4	2,723	9	HI
Russia	9	− 22	23	− 25	9	71,316	2092	UMI
USA	10	− 23	14	− 1	26	162,301	2441	HI
Austria	11	− 24	43	− 67	3	142,155	1,881	HI
Norway	12	− 26	17	− 16	8	72,550	240	HI
Taiwan	13	− 27	26	− 26	44	713	36	HI
Sweden	14	− 29	34	− 50	4	124,632	1451	HI
Germany	15	− 31	41	− 57	38	85,767	1343	HI
Syria	16	− 33	34	− 50	2	2,272	131	LI
Morocco	17	− 38	9	8	2	25,711	396	LMI
Turkey	18	− 39	27	− 33	5	111,113	965	UMI
Colombia	19	− 42	4	40	11	99,422	2505	UMI
South Africa	20	− 45	44	− 69	2	57,740	1522	UMI
Bulgaria	21	− 47	18	− 17	6	110,161	4564	UMI
Czechia	22	− 47	32	− 44	4	235,919	3443	HI
India	23	− 47	3	96	17	24,599	340	LMI
Serbia	24	− 48	20	− 21	7	189,090	1850	UMI
Philippines	25	− 48	5	30	5	24,611	446	LMI
Argentina	26	− 49	1	151	16	124,245	2575	UMI
Ethiopia	27	− 50	45	− 75	2	3,407	56	LI
Peru	27	− 50	10	0	2	67,455	5953	UMI
Belgium	29	− 50	10	0	4	180,624	2431	HI
Pakistan	30	− 54	30	− 38	2	5,495	123	LMI
Poland	31	− 55	34	− 50	2	103,073	2435	HI
Albania	32	− 57	6	25	4	73,962	1132	UMI
Mexico	33	− 57	8	14	8	31,213	2348	UMI
Greece	34	− 57	42	− 63	23	116,597	2002	HI
Venezuela	35	− 59	31	− 42	8	15,711	188	UMI
Denmark	36	− 61	33	− 46	9	136,410	555	HI
Italy	37	− 62	16	− 9	33	103,759	2327	HI
Spain	38	− 64	47	− 88	7	132,358	1880	HI
Brazil	39	− 65	15	− 8	3	103,532	2876	UMI
Paraguay	40	− 66	6	25	2	68,739	2452	UMI
Netherlands	41	− 68	21	− 22	4	179,544	1196	HI
Romania	42	− 69	27	− 33	15	92,012	2989	HI
Egypt	43	− 71	23	− 25	16	3,474	196	LMI
Canada	44	− 71	2	125	2	57,828	788	HI
Uruguay	45	− 72	34	− 50	2	120,773	1803	HI
Kenya	46	− 75	10	0	2	5461	100	LMI
Slovakia	47	− 79	34	− 50	2	242,951	2948	HI
Average		− 44		− 18		83,955	1537	
Median		− 48		− 25		73,962	1451	

This table shows the average percentage change in the number of surgeries participated/scrubbed in and the number of seminars attended between prior to and during the COVID-19 pandemic in each country. Countries with fewer than two respondents are excluded. Ranking of countries was based on percentages with up to five decimal points. Shown percentages are rounded to full percentage points. Countries are sorted based on reported change in surgeries residents participated/scrubbed in. Each country’s COVID-19 cases and COVID-19-related deaths per million through 2021 were obtained from the Institute for Health Metrics and Evaluation (IHME) [24]. Income classification comes from the World Bank [25]. The average and median are obtained across countries

The median percentage change during the pandemic is four and seven percentage points closer to zero than the corresponding averages in surgeries (− 48% versus − 44%) and seminars (− 25% versus − 18%), respectively. This suggests that a limited number of countries had negligible or even positive percentage change in learning inputs during the pandemic, while the preponderance of nations experienced substantial declines in resident training inputs.

### Learning Outputs

**Fig. 3:**
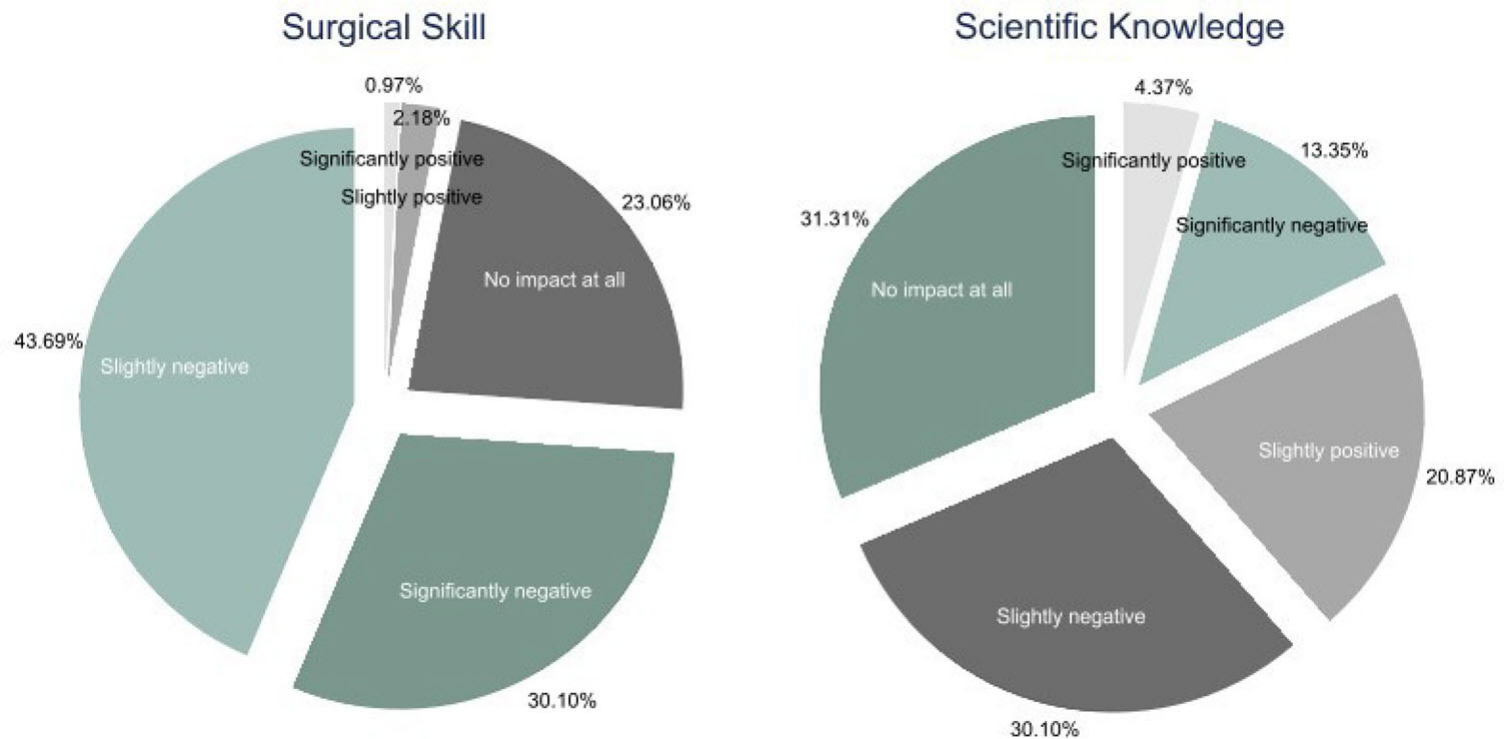
Reported Change in Surgical Skill and Scientific Knowledge of Plastic Surgery Residents due to COVID-19. *Notes:* These figures show the percentage of respondents reporting changes in their surgical skill (left) and scientific knowledge (right) due to COVID-19.

Figure 3 plots the survey responses regarding the impact of COVID-19 on residents’ skill and scientific knowledge. We find that 74% of the residents report a slightly negative (43.69%) or significantly negative (30.10%) impact on their surgical skill. In contrast, the scientific knowledge of plastic surgery residents was relatively preserved with 43.45% claiming slight or significant losses in their scientific knowledge attributed to the pandemic.

**Fig. 4:**
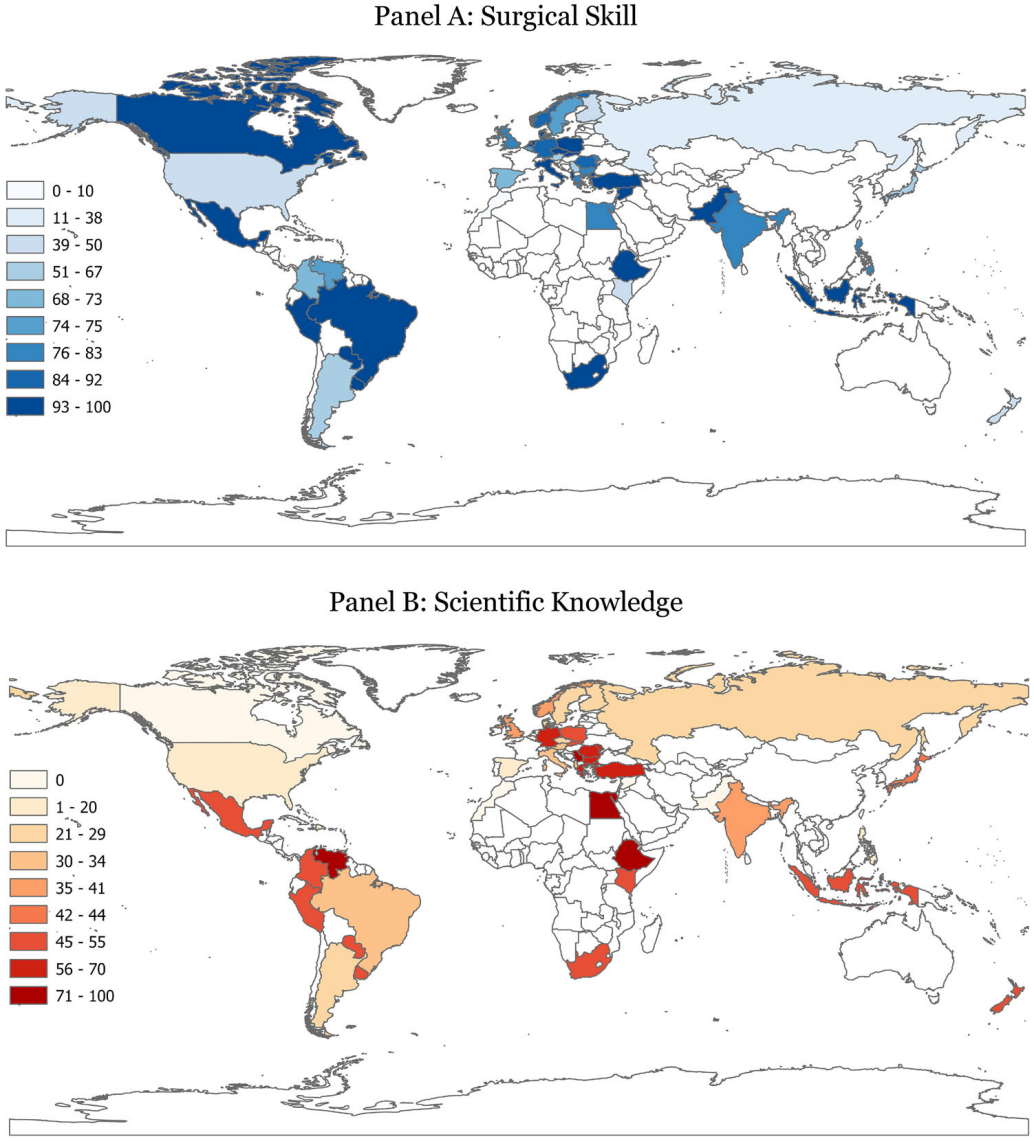
Reported loss in learning outputs due to COVID-19 by country. Panel A: surgical skill. Panel B: scientific knowledge

Table 3 shows the percentage of respondents in each country reporting slightly or significantly negative impact on their surgical skill and scientific knowledge due to the pandemic (also shown in Figure 4). Residents in the Dominican Republic, Russia, the USA, and Taiwan are the least likely to report surgical skill losses due to COVID-19. On the other extreme, residents from Turkey, the Netherlands, Mexico, Italy, Albania, Brazil, Czech Republic, and Denmark were the most likely to report a negative COVID-19 impact on their surgical dexterity. Respondents from Morocco were the only ones who did not report any surgical skill loss. Our analysis reveals a portion of countries in which residents cruised through the pandemic with their scientific knowledge intact. Six countries, Belgium, Canada, Morocco, the Netherlands, Pakistan, and Syria, had zero percent of trainees reporting scientific knowledge loss due to COVID-19. At the same time, Ethiopia, Venezuela, Serbia, and Egypt were the most heavily impacted with respect to the scientific knowledge of their trainees.

**Table 3:** Losses in surgical skills and scientific knowledge due to COVID-19 by country

Country	Rank	%	Rank	%	N	COVID-19 cases (per million)	COVID-19 deaths (per million)	Income
Morocco	1	0	1	0	2	25,711	396	LMI
Dominican Rep.	2	10	7	10	10	37,295	378	UMI
Russia	3	44	11	22	9	71,316	2092	UMI
USA	4	46	9	19	26	162,301	2441	HI
Finland	5	50	13	25	4	48,880	309	HI
Taiwan	5	50	21	36	44	713	36	HI
South Korea	5	50	25	42	12	12,260	109	HI
Kenya	5	50	27	50	2	5461	100	LMI
New Zealand	5	50	27	50	4	2723	9	HI
Serbia	10	57	45	86	7	189,090	1850	UMI
Argentina	11	63	13	25	16	124,245	2575	UMI
Austria	12	67	18	33	3	142,155	1881	HI
Japan	12	67	26	44	9	13,984	148	HI
Spain	14	71	8	14	7	132,358	1880	HI
Colombia	15	73	37	55	11	99,422	2505	UMI
Belgium	16	75	1	0	4	180,624	2431	HI
Albania	16	75	13	25	4	73,962	1132	UMI
Sweden	16	75	13	25	4	124,632	1451	HI
Venezuela	16	75	46	88	8	15,711	188	UMI
Philippines	20	80	10	20	5	24,611	446	LMI
UK	20	80	23	40	5	191,647	2627	HI
Moldova	20	80	38	60	5	114,927	3139	LMI
Egypt	23	81	44	81	16	3474	196	LMI
India	24	82	24	41	17	24,599	340	LMI
Greece	25	83	43	70	23	116,597	2002	HI
Bulgaria	26	83	41	67	6	110,161	4564	UMI
Romania	27	87	41	67	15	92,012	2989	HI
Germany	28	87	40	66	38	85,767	1343	HI
Norway	29	88	22	38	8	72,550	240	HI
Canada	30	100	1	0	2	57,828	788	HI
Netherlands	30	100	1	0	4	179,544	1196	HI
Pakistan	30	100	1	0	2	5495	123	LMI
Syria	30	100	1	0	2	2272	131	LI
Denmark	30	100	11	22	9	136,410	555	HI
Czechia	30	100	13	25	4	235,919	3443	HI
Brazil	30	100	18	33	3	103,532	2876	UMI
Italy	30	100	18	33	33	103,759	2327	HI
Indonesia	30	100	27	50	2	15,473	523	LMI
Mexico	30	100	27	50	8	31,213	2348	UMI
Paraguay	30	100	27	50	2	68,739	2452	UMI
Peru	30	100	27	50	2	67,455	5953	UMI
Poland	30	100	27	50	2	103,073	2435	HI
Slovakia	30	100	27	50	2	242,951	2948	HI
South Africa	30	100	27	50	2	57,740	1522	UMI
Uruguay	30	100	27	50	2	120,773	1803	HI
Turkey	30	100	38	60	5	111,113	965	UMI
Ethiopia	30	100	47	100	2	3407	56	LI
Average		78		40		83,955	1537	
Median		82		41		73,962	1451	

This table shows the percentage of respondents in each country reporting slightly or significantly negative change in surgical skill or scientific knowledge due to the COVID-19 pandemic. Countries with fewer than two respondents are excluded. Ranking of countries was based on percentages with up to five decimal points. Shown percentages are rounded to full percentage points. Countries are sorted based on reported surgical skill loss. Each country’s COVID-19 cases and COVID-19-related deaths per million through 2021 were obtained from the Institute for Health Metrics and Evaluation (IHME) [24]. Income classification comes from the World Bank [25]. The average and median are obtained across countries.

We find that the median losses level is four and one percentage point higher than the corresponding averages in surgeries (82% versus 78%) and scientific knowledge (41% versus 40%), respectively. This suggests that a limited number of countries had limited or negligible impact of COVID-19 on residents’ surgical skill, while the preponderance of nations reported sizable losses in surgical skill.

We investigate the statistical association between changes in surgeries and seminars during COVID-19 and the share of respondents reporting declined surgical skill (*ρ* = *−*0*.*511 with *p <* 0*.*001 for surgeries; *ρ* = *−*0*.*079 with *p* = 0*.*600 for seminars) and decreased scientific knowledge (*ρ* = *−*0*.*118 with *p* = 0*.*428 for surgeries; *ρ* = *−*0*.*274 with *p* = 0*.*006 for seminars). It is important to note the significant correlations between corresponding input and output (i.e., surgeries scrubbed in with surgical skill). We find weak cross-correlations between inputs and outputs (i.e., surgeries attended with scientific knowledge and seminars attended with surgical skill). This suggests limited substitutability of inputs in training. In other words, it may be challenging to make up for lost operating room time with more seminars.

### COVID-19 Pandemic Burden

The COVID-19 burden of disease may reflect pressures to each country’s healthcare system and is likely to have influenced disruptions in resident training during the pandemic. Tables 2 and 3 report the pandemic-related burden of disease in each country next to the corresponding pandemic-related change in learning inputs and outputs, respectively.

We investigate the association between pandemic-induced changes in plastic surgery residents’ learning inputs and outputs, and the number of COVID-19 cases and COVID-19-related deaths through the end of 2021. We find substantial correlations between COVID-19 prevalence per country until the end of 2021 and the percentage change in the number of surgeries residents scrubbed in during COVID-19 (*ρ* = *−* 0*.*188 with *p* = 0*.*206 for cases; *ρ* = 0*.*202 with *p* = 0*.*174 for deaths). We find limited association between COVID-19 prevalence and the percentage change in seminars residents attended during the pandemic in each country (*ρ* = *−*0*.*056 with *p* = 0*.*709 for cases; *ρ* = 0*.*077 with *p* = 0*.*605 for deaths). Turning to learning outputs, we find sizable correlations between COVID-19 prevalence per country until the end of 2021 and the share of respondents reporting declined surgical skill (*ρ* = 0*.*207 with *p* = 0*.*163 for cases; *ρ* = 0*.*2840 with *p* = 0*.*053 for deaths). The correlation of COVID-19 disease burden and reported decreased scientific knowledge is found to be relatively weak (*ρ* = *− *0*.*094 with *p* = 0*.*530 for cases; *ρ* = 0*.*120 with *p* = 0*.*422 for deaths).

### Regional Analysis

Tables 4 and 5 show the COVID-19 impact on learning inputs (outputs) by geographical region. South America reports the largest decline in surgeries and surgical skill during the pandemic compared with other regions. Trainees in Europe, Asia, and Africa follow in terms of surgical skill losses. Plastic surgery residents in North America and Oceania seem to be the least affected by the pandemic. Even at the regional level, we observe smaller losses in areas with lower input declines (e.g., residents from North America report attending 28% more seminars during the pandemic and only 20% of them claim a negative COVID-19 impact on their scientific background).

**Table 4:** Changes in surgeries and seminars attended during COVID-19 by region

Region	Reported change in		N	Countries	COVID-19 cases (per million)	COVID-19 deaths (per million)
	Surgeries (%)	Seminars				
Africa	− 56	− 32	24	5	19,159	454
Asia	− 31	− 15	107	10	28,184	491
Europe	− 46	− 34	187	20	133,851	2037
North America	− 35	28	46	4	72,159	1489
Oceania	− 19	− 76	4	1	2723	9
South America	− 57	17	44	7	85,697	2622

This table shows the average percentage change in the number of surgeries participated/scrubbed in and the number of seminars attended between prior to and during the COVID-19 pandemic in each region. Countries with fewer than two respondents are excluded. Each country’s COVID-19 cases and COVID-19-related deaths per million through 2021 were obtained from the Institute for Health Metrics and Evaluation (IHME) [24].

**Table 5:** Losses in surgical skills and scientific knowledge due to COVID-19 by region

Region	Reported loss in		N	Countries	COVID-19 cases (per million)	COVID-19 deaths (per million)
	Surgical skill	Scientific knowledge				
	%	%				
Africa	66	56	24	5	19,159	454
Asia	77	32	107	10	28,184	491
Europe	83	40	187	20	133,851	2037
North America	64	20	46	4	72,159	1489
Oceania	50	50	4	1	2723	9
South America	87	50	44	7	85,697	2622

This table shows the percentage of respondents in each region reporting slightly or significantly negative change in surgical skill or scientific knowledge due to the COVID-19 pandemic. Countries with fewer than two respondents are excluded. Each country’s COVID-19 cases and COVID-19-related deaths per million through 2021 were obtained from the Institute for Health Metrics and Evaluation (IHME) [24].

### World Bank Income Classification

Table 6 shows the percentage change in surgeries and seminars by respondents from countries in each World Bank income-level classification. We find that the decline in reported surgeries was roughly 40%, on average, across all four income classifications, suggesting a ubiquitous impact of COVID-19 on operating volume across countries regardless of their income. In contrast, we find varying levels of COVID-19 impact on the seminars trainees attended. Residents from low-income countries reported the most dramatic negative impact on seminars (i.e., more than 60% decrease). Table 7 shows the percentage of respondents reporting negative impact on their surgical skill and scientific knowledge due to the pandemic in countries in each World Bank income-level classification. Residents from low-income countries stand out, as 100% of them report a negative impact in their surgical skill and half of them report a decline in their scientific knowledge as a result of COVID-19.

**Table 6:** Changes in surgeries and seminars attended during COVID-19 by income Level

Income level	Reported change in		N	Countries	COVID-19 cases (per million)	COVID-19 deaths (per million)
	Surgeries	Seminars				
	%	%				
High Income (HI)	− 43	− 30	264	23	111,281	1539
Upper Middle Income (UMI)	− 45	1	93	14	82,928	2243
Lower Middle Income (LMI)	− 44	− 4	51	8	27,469	658
Low Income (LI)	− 42	− 63	4	2	2,840	94

This table shows the average percentage change in the number of surgeries participated/scrubbed in and the number of seminars attended between prior to and during the COVID-19 pandemic in countries in each income level. Countries with fewer than two respondents are excluded. Each country’s COVID-19 cases and COVID-19-related deaths per million through 2021 were obtained from the Institute for Health Metrics and Evaluation (IHME) [24]. Income classification comes from the World Bank [25].

**Table 7:** Losses in surgical skills and scientific knowledge due to COVID-19 by income level

Income level	Reported loss in		N	Countries	COVID-19 cases (per million)	COVID-19 deaths (per million)
	Surgical skill	Scientific knowledge				
	%	%				
High Income (HI)	79	35	264	23	111,281	1539
Upper Middle Income (UMI)	77	48	93	14	82,928	2243
Lower Middle Income (LMI)	72	38	51	8	27,469	658
Low Income (LI)	100	50	4	2	2840	94

This table shows the percentage of respondents from countries in each income level reporting slightly or significantly negative change in surgical skill or scientific knowledge due to the COVID-19 pandemic. Countries with fewer than two respondents are excluded. Each country’s COVID-19 cases and COVID-19-related deaths per million through 2021 were obtained from the Institute for Health Metrics and Evaluation (IHME) [24]. Income classification comes from the World Bank [25].

## Discussion

Our results show that the preponderance of plastic surgery residents across the world expects their surgical skill and scientific knowledge to be lower compared with previous cohorts due to the pandemic. Residents are much more likely to report surgical skill losses than scientific knowledge losses (i.e., 73.79% versus 43.45%). At the same time, pockets of residents may have experienced positive consequences from COVID-19 on personal and professional dimensions, such as trauma and emergency case care [14, 17]. Roughly one-third (31.31%) of residents reported no COVID-19 impact on their scientific knowledge, while more than one-fifth (20.87%) experienced a positive impact from the pandemic. Some residents might have been able to invest time in self-study or research during the pandemic, preserving their knowledge capital from depreciation [18, 31–33].

Digital resources, such as video recordings of operations, webinars, and teleconferences, might have also benefited residents’ scientific knowledge [18, 34]. Remedial measures that target surgical skill may be more needed than those targeting scientific knowledge. Potential remedial strategies include a surgical skills laboratory or simulating surgical procedures on practice models [35, 36].

Our results corroborate previous studies on the COVID-19 impact on the surgical logged hours of residents across surgical specialties at the national and regional level [6, 37, 38]. This suggests that our findings on learning outputs of plastic surgery residents may constitute a benchmark for the pandemic-related learning losses of residents more broadly [39–44].

Our finding that surgical skill is primarily driven by the surgeries residents scrub in and less so by seminars provides clear guidance regarding the necessary remedial strategies. Program directors, health policy makers, and health system administrators can leverage our findings into designing recovery plans that provide residents with operation room exposure to help mitigate their pandemic-induced learning losses [45].

Our results support the need for flexible training models, competency-based advancement, and regular assessment of trainees. This training approach may be best suited to mitigate crisis-driven training deficits [1]. National associations such as the American Board of Plastic Surgery and the Accreditation Council for Graduate Medical Education can lead the efforts to design effective recovery plans for plastic surgery residents [46].

Our study brings forth two contextual benchmarks of the impact of COVID-19 on the plastic surgery residents in each country: the COVID-19-related disease burden and the income level. Countries with substantial learning losses and a COVID-19 caseload close to the average (e.g., Mexico) may experience low system resilience more generally [47].^2^ These countries may need to invest in fortifying their healthcare system (e.g., through resource redundancy) and in the learning recovery of their residents to ensure they become effective health professionals.

Each country’s income level may be correlated with resource availability that would make healthcare systems and training programs more resilient to crises and more likely to bounce back after a crisis. Residents in high-income countries like Japan, the UK, South Korea, and New Zealand, who suffered lower loss levels, may have better access to tools that would help them recover compared with their counterparts in less affluent countries.

This study has certain limitations. First, our focus on plastic surgery trainees limits the statistical power of our analyses. Future studies can potentially reach residents across multiple specialties. Second, our convenient non-random sample of participants represents a broad geographic distribution of resident experiences during the pandemic. At the same time, the severity of pandemic may influence the likelihood of plastic surgery residents from specific countries to participate in the study. Future studies might be able to capture the pandemic-related training disruptions of plastic surgery residents in countries or communities not reachable at this time.

Third, our self-reported measures of learning inputs and anticipated outputs may contain recall and expectation bias. Further research could explore the availability of administrative data on log books and board examinations scores to measure training inputs and outputs and obtain more accurate estimates of the COVID-19 impact.

At the same time, this study can serve as a blueprint for future research on resident training inputs and outputs, especially following system-wide shocks. The COVID-19 pandemic impaired resident training across the world. Future epidemics, natural phenomena associated with climate change, geopolitical instabilities also pose a significant threat to resident training in the future [48–50]. Our study contributes to a broader understanding of the resilience of each country’s resident training programs to crises. Our approach to quantifying resident learning inputs and expected outputs is general and can be applied in more contexts and specialties. Our measures of learning outputs, in particular, speak to current proposals to develop a “Surgical Preparedness Index” (SPI) to assess, monitor, and improve the resilience of training programs and healthcare systems across the world [48].

## Supplementary Information

Below is the link to the electronic supplementary material.

Supplementary file1 (DOCX 372 KB)

## Footnotes

1 The average survey completion rate for those was less than 30%.

2 A low death toll combined with high learning losses in a country may be indicative of sacrificed training to save lives.
