# Supplementary material for: The Impact of COVID-19 on Plastic Surgery Residents Across the World: A Country-, Region-, and Income-level Analysis
Source: Aesthetic Plast Surg. 2023 May 30;47(6):2889–901. doi: 10.1007/s00266-023-03389-w (PMC10228894; doi:10.1007/s00266-023-03389-w)
Supplement: Supplementary file 1 — Supplementary file1 (DOCX 372 KB) [file 266_2023_3389_MOESM1_ESM.docx]

**Supplementary Appendix**

**The Impact of COVID-19 on Plastic Surgery Residents Across the World: A Country-, Region-, and Income-level Analysis**

**S1 Survey Design**

The survey was designed after a comprehensive review of the literature. The survey instrument was refined by an expert panel with experience in survey methodology and administration, and plastic surgery education. Consensus agreement among the researchers’ panel resulted in drafting of a primary instrument. The draft instrument was piloted in a soft launch within two major academic plastic surgery clinics. After incorporation of the valuable feedback obtained, the questionnaire was finalized.

# S2 Survey Dissemination

The survey was conducted on Qualtrics (Qualtrics Provo, UT). The survey induction letter with the survey link was emailed by the International Society for Aesthetic Plastic Surgery (ISAPS) to national associations and societies of plastic surgeries that consists its global alliance. Additional national associations of plastic surgeons were contacted directly by the researchers. Figure [S1](#_bookmark63) shows the cohorts of pandemic-impacted plastic surgery residents targeted by the survey. There is no information on how the mailing lists of resident members in national and international societies of plastic surgery residents get updated with older cohorts being removed and newer cohorts being added. Thu, we make a conservative assumption that at least 50% of resident members in the mailing lists were in a plastic surgery program during the pandemic. This facilitates the calculation of a pre-data collection potential sample calculation.

Figure S1: Targeted Resident Population


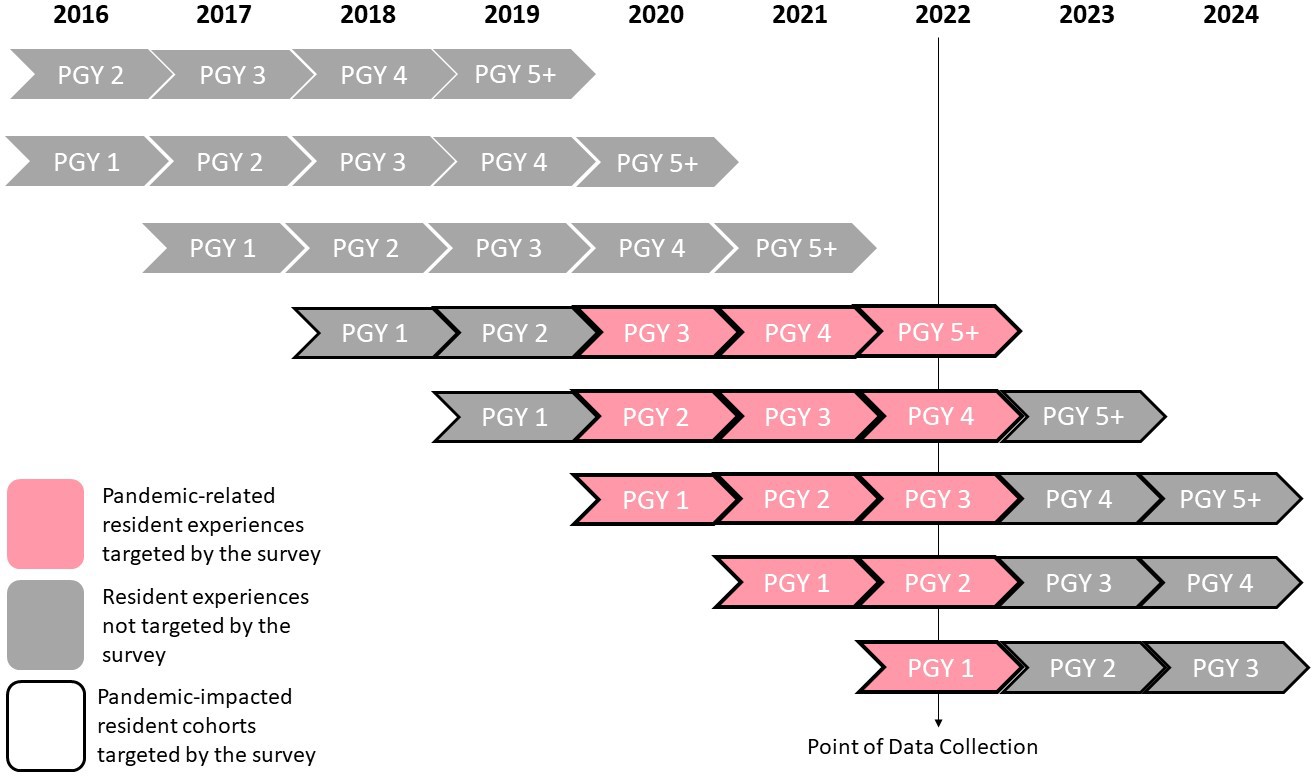


*Notes: This graphic shows the cohorts of pandemic-impacted plastic surgery residents targeted by the survey. As the mailing lists of resident members in national and international societies of plastic surgery residents got updated in early 2022 with older cohorts being removed and newer cohorts being added, a conservative estimate of at least 50% of resident members in 50% being in a plastic surgery program during the pandemic facilitates the calculation of a pre-data collection potential sample calculation.*

In total, 63 national or international associations and societies of plastic, reconstructive, and aesthetic surgeons around the globe were invited to disseminate the survey link to their resident members. These are:

1. ARGENTINA - Sociedad Argentina de Cirugia Plastica Estetica y Reparadora (SACPER)
2. AUSTRALIA - Australasian Society of Aesthetic Plastic Surgery (ASAPS)
3. AUSTRIA -

Chirurgie (OGPA¨ RC)

O¨ sterreichische Gesellschaft fu¨r Plastische, Asthetische und Rekonstruktive

1. AZERBAIJAN - Society of Plastic Surgery Azerbaijan (SPSA)
2. BELGIUM - Royal Belgian Society for Plastic Surgery (RBSPS)
3. BOLIVIA - Sociedad Boliviana de Cirugia Plastica Estetica y Reparadora (SBCPER)
4. BRAZIL - Sociedade Brasileira de Cirurgia Pl´astica (SBCP)
5. CANADA - Canadian Society for Aesthetic Plastic Surgery (CSAPS)
6. CHILE - Sociedad Chilena de Cirug´ıa Pl´astica, Reconstructiva y Est´etica (SCCPRE)
7. CHINA - Chinese Society of Plastic Surgery (CSPS)
8. CHINESE TAIPEI - Taiwan Society of Plastic Surgery (TSPS)
9. COLOMBIA - Sociedad Colombiana de Cirug´ıa Pl´astica, Est´etica y Reconstructiva (SCCP)
10. CYPRUS - Cyprus Society of Plastic, Reconstructive and Aesthetic Surgery (CySPRAS)
11. CZECH REPUBLIC - Czech Society of Aesthetic Surgery (CSAS)
12. CZECH REPUBLIC - Czech Society of Plastic Surgery (CSPS)
13. DENMARK - Dansk Selskab for Kosmetisk Plastikkirurgi (DSKP)
14. DOMINICAN REPUBLIC - Sociedad Dominican de Cirug´ıa Plastica Reconstructiva y Est´etica (SODOCIPRE)
15. EASAPS - European Association of Societies of Aesthetic Plastic Surgery (EASAPS)
16. ECUADOR - Sociedad Ecuatoriana de Cirug´ıa Pl´astica, Reconstructiva y Est´etica (SECPRE)
17. EGYPT - Egyptian Society of Plastic and Reconstructive Surgeons (ESPRS)
18. FINLAND - Suomen Esteettiset Plastikkakirurgit (SEP)
19. FRANCE - Societ´e Fran¸caise des Chirurgiens Esth´etiques Plasticiens (SOFCEP)
20. GERMANY - Vereinigung der Deutschen Aesthetisch Plastischen Chirurgen (VDAPC)
21. GREECE - Hellenic Society of Plastic, Reconstructive and Aesthetic Surgery (HESPRAS)
22. INDIA - Indian Association of Aesthetic Plastic Surgeons (IAAPS)
23. INDONESIA - Indonesian Association of Plastic Reconstructive and Aesthetic Surgeons (InaPRAS)
24. IRAN - Iranian Society of Plastic and Aesthetic Surgeons(ISPAS)
25. IRELAND - Irish Association of Plastic Surgeons (IAPS)
26. ITALY - Associazione Italiana di Chirurgia Plastica Estetica (AICPE)
27. ITALY - Societ`a Italian di Chirurgia Plastica Ricostruttiva ed Estetica (SICPRE)
28. JAPAN - Japan Society of Aesthetic Plastic Surgery (JSAPS)
29. LEBANON - Lebanese Society of Plastic, Reconstructive, and Aesthetic Surgery (LSPRAS)
30. MALAYSIA - Malaysian Association of Plastic, Aesthetic and Craniomaxillofacial Surgeons (MAPACS)
31. MEXICO - Asociaci´on Mexican de Cirug´ıa Pl´astica Est´etica y Reconstructiva (AMCPER)
32. NETHERLANDS - Nederlandse Vereniging voor Esthetische Plastische Chirurgie (NVEPC)
33. NORWAY - Norwegian Society of Aesthetic Plastic Surgery (NSAP)
34. OSAPS - Oriental Society of Aesthetic Plastic Surgery (OSAPS)
35. PAKISTAN - Pakistan Association of Plastic Surgeons (PAPS)
36. PANAMA - Asociacion Panamen˜a de Cirugia Plastica, Estetica y Reconstructiva (APCPER)
37. PERU - Sociedad Peruana de Cirug´ıa Pl´astica (SPCP)
38. PHILIPPINES - Philippine Association of Plastic, Reconstructive and Aesthetic Surgeons (PAPRAS)
39. PORTUGAL - Sociedade Portuguesa de Cirurgia Pl´astica Reconstrutiva e Est´etica (SPCPRE)
40. ROMANIA - Romanian Aesthetic Surgery Society (RASS)
41. RUSSIA - Northeastern Society of Plastic and Reconstructive Surgeons (NESPRS)
42. SERBIA - Serbian Society of Aesthetic Plastic Surgeons (SRBSAPS)
43. SERBIA - Serbian Society of Plastic, Reconstructive, and Aesthetic Surgery (SRBPRAS)
44. SINGAPORE - Singapore Association of Plastic Surgeons (SAPS)
45. SOUTH AFRICA - Association of Plastic, Reconstructive and Aesthetic Surgeons of South- ern Africa (APRASSA)
46. SOUTH KOREA - Korean Society of Aesthetic Plastic Surgery (KSAPS)
47. SPAIN - Asociaci´on Espan˜ola de Cirug´ıa Est´etica Pl´astica (AECEP)
48. SPAIN - Sociedad Espan˜ola de Cirug´ıa Pl´astica Reparadora y Est´etica (SECPRE)
49. SWEDEN - Svensk F¨orening f¨or Estetisk Plastikkirurgi (SFEP)
50. SWITZERLAND - Schweizerische Gesellschaft fu¨r Aesthetische Chirurgie (SGAC)
51. SWITZERLAND - Swiss Society of Plastic, Reconstructive and Aesthetic Surgery (SSPRAS)
52. THAILAND - Society of Aesthetic Plastic Surgeons of Thailand (THSAPS)
53. TURKEY - Turkish Society of Aesthetic Plastic Surgery (TSAPS)
54. UNITED KINGDOM - British Association of Aesthetic Plastic Surgeons (BAAPS)
55. UNITED KINGDOM - United Kingdom Association of Aesthetic Plastic Surgeons (UKAAPS)
56. UNITED STATES - American Society for Aesthetic Plastic Surgery, Inc. (ASAPS)
57. VENEZUELA - Sociedad Venezolana de Cirug´ıa Pl´astica, Reconstructiva, Est´etica y Max- ilofacial

(SVCPREM)

1. VIETNAM - Vietnamese Society of Aesthetic and Plastic Surgery (VSAPS)
2. GLOBAL - ICOPLAST (International Confederation of Plastic Surgery Societies)
3. GLOBAL - International Society of Aesthetic Plastic Surgery (ISAPS)

Emails were sent on the 10^th^, 17^th^, 24^th^ and 31^st^ of January from ISAPS to its resident members specifically about our study. On the 19^th^, 26^th^ of January and on the 2^nd^ of February resident members of ISAPS were reminded of the study via the weekly ISAPS-NEWS email.

There was rigorous dissemination of the survey through social media. The survey link and the survey’s flyer was posted in the official Instagram accounts of ICOPLAST (International Confeder- ation of Plastic Surgery Societies), ISAPS, PRS Interest Group India, LPRSIG (Lebanese Plastic and Reconstructive Surgery Interest Group), Yehandsurgeons (Young Europeans Hand Surgeons), IPSRG (International Plastic Surgery Organization), UBMS PRAS Society (University of Buck- ingham Plastic, Reconstructive & Aesthetic Surgery Society), BAAPS (The British Association of Aesthetic Plastic Surgeons), CGSTA (Canadian Global Surgery Trainee Alliance).

**Survey Questions**

**Start of Block: Default Question Block**

Q1 You are invited to participate in a research study on the **impact of the COVID-19 pandemic on the Residency Training of Plastic Surgeons**. According to the World Health Organization, the COVID-19 pandemic period started on March 11th, 2020. You will be asked to answer a series of questions. Please provide honest responses. Your participation will take approximately 8 minutes.

***No risks are associated with your participation or not in the study.*** We cannot and do not guarantee or promise that you will receive any benefits from this study.

If you have read this form and have decided to participate in this project, please understand your participation is voluntary and you have the right to withdraw your consent or discontinue participation at any time without penalty or loss of benefits to which you are otherwise entitled. The alternative is not to participate. You have the right to refuse to answer particular questions. The results of this research study may be presented at scientific or professional meetings or published in scientific journals. Your individual privacy will be maintained in all published and written data resulting from the study.

Identifiers might be removed from identifiable private information and, after such removal, the information could be used for future research studies or distributed to another investigator for future research studies without additional informed consent from you. If you provide your contact information, your contact information will be linked to your responses and we may reach out to you about a possible follow-up related to this study.

This research is conducted in collaboration with the **University of Ioannina** and **Stanford University**, under the auspices of the **International Society of Aesthetic Plastic Surgery (ISAPS)**. If you have any questions, concerns or complaints about this research, its procedures, risks and benefits, contact the Protocol Director, Sofoklis Goulas, Ph.D. at [goulas@stanford.edu.](mailto:goulas@stanford.edu)

If you are not satisfied with how this study is being conducted, or if you have any concerns, complaints, or general questions about the research or your rights as a participant, please contact the Stanford Institutional Review Board (IRB) to speak to someone independent of the research team at 650-723-2480 or email at [irbnonmed@stanford.edu](mailto:irbnonmed@stanford.edu) (for nonmedical studies),

or toll free at 1-866-680-2906. You can also write to the Stanford IRB, Stanford University, 1705 El Camino Real, Palo Alto, CA 94306.

We will collect your personal data for this study, including data related to your health and/or other sensitive personal data as described in this consent form. We refer to this data as “Your Study Data.” Any Study Data from this research that is conducted in the European Union/European Economic Area (EU/EEA) will be collected, stored, used, and shared (processed) as required by the EU/EEA law known as the General Data Protection Regulation (“GDPR”). Your Study Data may be processed for the following reasons (purposes): - to carry out and confirm the accuracy of the study; - to help us monitor and ensure that the study is following research best practices; - to make required reports to domestic and foreign regulatory agencies and government officials who have a duty to monitor and oversee studies like this one; and, - to follow applicable laws and regulations, including requirements that data from this study, without information that could directly identify you, be made available to other researchers not affiliated with the study sponsor or the study team. For example, regulatory authorities in some countries may require that Your Study Data, without information that could directly identify you, be made publicly available on the internet or in other ways, to make research data more widely available to other researchers. The following persons and organizations may process Your Study Data for the purposes listed above: - the study team, including other people who, and organizations that, assist the study team: Georgios Karamitros, MD (georgios.karamitros@ldh.nhs.uk) and Sofoklis Goulas, Ph.D. (goulas@stanford.edu). - the ethics committee or institutional review board that approved this study; and - domestic and foreign regulatory agencies and government officials who have a duty to monitor or oversee studies like this one.

We may conduct the study in the United States and other countries where the laws do not protect your privacy to the same extent as the laws in the country where you live (reside). Your Study Data may be transferred to these countries for the purposes described above. We will take reasonable steps to protect your privacy, consistent with applicable laws. For example, where appropriate, we enter into data transfer agreements with standard contractual clauses approved by European authorities that provide certain terms and conditions on how Your Data can be used and shared. These data transfer agreements help ensure Your Study Data is adequately protected.

The GDPR gives you certain rights with regard to Your Study Data, including the right to: (1) request access to, correct, or erase Your Study Data, (2) object to or restrict our processing of Your Study Data, and (3) request that we move, copy or transfer Your Study Data to another organization. To make any such requests, please contact the Principal Investigator at

[goulas@stanford.edu.](mailto:goulas@stanford.edu) You may also withdraw your consent at any time. If you withdraw your consent or request Your Study Data be erased, we can still legally collect, use and share Your Study Data up to the point in time that you withdraw your consent or request your data be erased. Even if you withdraw your consent, we may still use Your Study Data that has been anonymized by removing any data that identifies you. We may also use and share Your Study Data that has been pseudonymized by removing your name and certain other identifiers so that the data does not directly identify you, where permitted by law. Your anonymized or pseudonymized data may be used for purposes of: (a) public health (e.g., ensuring the high quality and safety of health care and/or of medical drugs or devices), (b) scientific or historical research or statistical analysis as allowed by the EU or EU Member State laws, and (c) saving or storing for important reasons of public interest. We will keep Your Study Data in identifiable form if required by law. There is no limit on the length of time we will keep Your Study Data for this research because it may be analyzed for many years. We will also keep your Study Data to follow our legal and regulatory requirements. We will keep it as long as it is useful, unless you decide you no longer want to take part. You are allowing access to this information indefinitely as long as you do not withdraw your consent. You consent to the collection, use and transfer of Your Study Data, which includes health and other sensitive personal data, for the purpose of carrying out the research study and know that you can withdraw your consent at any time, and we will stop processing your personal data, except as described above.

Please print a copy of this page for your records.

If you agree to participate in this research, please select "I agree."

- I agree (1)
- I do not agree (2)

*Skip To: End of Survey If You are invited to participate in a research study on the impact of the COVID-19 pandemic on the... = I do not agree*

*Skip To: Q1 If You are invited to participate in a research study on the impact of the COVID-19 pandemic on the... = I agree*

Q1 How old are you?

20 25 30 35 40 45 50 55 60


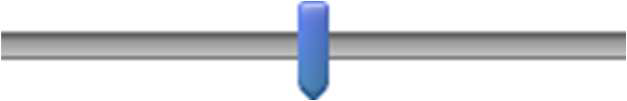


Years ()

Q2 What is your gender?

- Male (1)
- Female (2)
- Non-binary / third gender (3)
- Prefer not to say (4)

Q3 Which of the following races do you consider yourself to be?

- White or Caucasian (1)
- Black or African American (2)
- American Indian or Alaska Native (3)
- Asian (4)
- Native Hawaiian or Pacific Islander (5)
- Multi-racial (7)
- Other (specify) (6)

Q4 Do you have dependents?

- Yes (1)
- No (2)
- Prefer not to say (3)


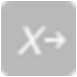


Q5 Which country is your specialty training program based in?

▼ Afghanistan (1) ... Zimbabwe (1357)

*Skip To: Q6 If List of Countries = United States of America*

*Skip To: Q7 If List of Countries != United States of America*

Q6 Which state is your specialty training program based in?

▼ Alabama (1) ... I do not reside in the United States (53)

Q7 Have you completed your primary medical degree in the same country your specialty training program is based in?

- Yes (1)
- No (2)

Q8 What kind of hospital provides your specialty training program?

- Community Hospital (2)
- Military Hospital (3)
- Private Hospital (5)
- Tertiary Hospital (1)
- University Hospital (4)

Q9 Does the entirety of your specialty training in Plastic Surgery get completed in a **single**

hospital?

- Yes (1)
- No (2)

Q10 How much training in *General Surgery* have you completed prior to the commencement of your specialty training in Plastic Surgery?

- Less than a year (1)
- Between 1 and 2 years (2)
- Between 2 and 4 years (3)
- More than 4 years (4)

Q11 Have you had *postgraduate experience in Plastic Surgery* prior to the commencement of your specialty training program in Plastic Surgery?

- Yes (1)
- No (2)

Q12 How long does your specialty training program in Plastic Surgery last?

- 1 year (1)
- 2 years (2)
- 3 years (3)
- 4 years (4)
- 5 years or more (5)

Q13 Does the hospital you work at treat COVID-19 patients?

- Yes (1)
- No (2)

*Skip To: Q15 If Does the hospital you work at treat COVID-19 patients? = No*

*Skip To: Q14 If Does the hospital you work at treat COVID-19 patients? = Yes*

Q14 Are you or have you been required to cover COVID-19 ward shifts during your specialty training program?

- Yes (1)
- No (2)

Q15 When did you start your specialty training in Plastic Surgery?

20122013201420152016201720182019202020212022


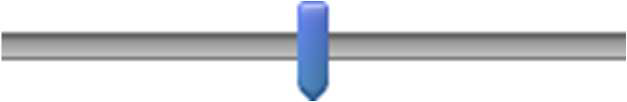


Year of specialty commencement ()

Q16 When are you estimating to complete your specialty training in Plastic Surgery?

2018201920202021202220232024202520262027202820292030


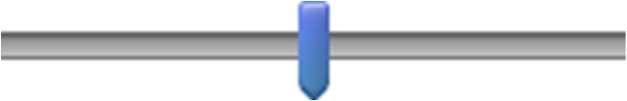


Year of completion ()

Q17 How many training seminars/courses/lectures were held weekly in your clinic **before the COVID-19 pandemic**?

- No seminar / course / lecture (6)
- 1 seminar / course / lecture per *month* (8)
- 2 seminars / courses / lectures per *month* (7)
- 1 seminar / course / lecture per *week* (1)
- 2 seminars / courses / lectures per *week* (2)
- 3 seminars / courses / lectures per *week* (3)
- 4 seminars / courses / lectures per *week* (4)
- 5 seminars / courses / lectures per *week* (5)

Q18 How many training seminars/courses/lectures were held weekly at your clinic **during the COVID-19 pandemic**?

- No seminar / course / lecture (6)
- 1 seminar / course / lecture per *month* (8)
- 2 seminars / courses / lectures per *month* (7)
- 1 seminar / course / lecture per *week* (1)
- 2 seminars / courses / lectures per *week* (2)
- 3 seminars / courses / lectures per *week* (3)
- 4 seminars / courses / lectures per *week* (4)
- 5 seminars / courses / lectures per *week* (5)

Q19 Has the number of surgeries you scrubbed in or participated in weekly decreased due to the COVID-19 pandemic?

- Yes (1)
- No (2)

Q20 How many surgeries **per week** did you scrub in/participate in as a resident **before the COVID-19 pandemic**?

0 2 4 6 8 10 12 14 16 18 20


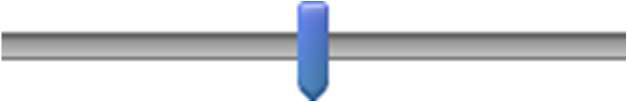


Number of surgeries per *week* ()

Q21 How many surgeries **per week** did you scrub in/participate in as a resident **during the COVID-19 pandemic**?

0 2 4 6 8 10 12 14 16 18 20


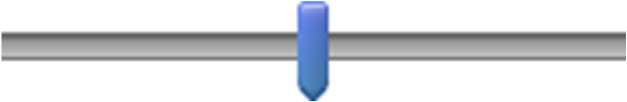


Number of surgeries per *week* ()

Q22 What has been the impact of the COVID-19 pandemic on your **scientific knowledge** in Plastic Surgery?

- Significantly negative (1)
- Slightly negative (2)
- No impact at all (3)
- Slightly positive (4)
- Significantly positive (5)

Q23 What has been the impact of the COVID-19 pandemic on your **surgical skills** in Plastic Surgery?

- Significantly negative (1)
- Slightly negative (2)
- No impact at all (3)
- Slightly positive (4)
- Significantly positive (5)
